# Relationship between CYP17A1 genetic polymorphism and coronary artery disease in a Chinese Han population

**DOI:** 10.1186/s12944-015-0007-4

**Published:** 2015-03-07

**Authors:** Chuan-Fang Dai, Xiang Xie, Yi-Ning Yang, Xiao-Mei Li, Ying-Ying Zheng, Zhen-Yan Fu, Fen Liu, Bang-Dang Chen, Min-Tao Gai, Yi-Tong Ma

**Affiliations:** Department of Cardiology, First Affiliated Hospital of Xinjiang Medical University, Urumqi, 830054 P.R. China

**Keywords:** CYP17A1, Single nucleotide polymorphism, Coronary artery disease, Case control study

## Abstract

**Background:**

CYP17A1 gene encodes P450c17 proteins, which is a key enzyme that catalyzes the formation of sex hormones. Many clinical studies showed that sex hormones levels play an important role in the pathogenesis of coronary artery disease (CAD). However, the relationship between CYP17A1 genetic polymorphisms and CAD remains unclear. The aim of this study was to investigate the association of CYP17A1 genetic polymorphisms with CAD in a Han population of China.

**Methods:**

A total of 997 people include 490 patients and 507 controls were selected for the present study. Five single-nucleotide polymorphisms (SNPs) (rs4919686, rs1004467, rs4919687, rs10786712, and rs2486758) were genotyped by using the real-time PCR (TaqMan) method.

**Results:**

For men, the rs10786712 was found to be associated with CAD in a recessive model (P = 0.016), after adjustment of the major confounding factors, the significant difference was retained (OR = 1.644, 95% confidence interval [CI]: 1.087-2.488, P = 0.019). For women, the rs1004467 was also found to be associated with CAD in a dominant model (P = 0.038), the difference remained statistically significant after multivariate adjustment (OR = 1.623, 95% CI: 1.023-2.576, P = 0.040). The distribution of rs4919687 genotypes showed a significant difference between CAD and control participants in a recessive model (P = 0.019), the significant difference was retained after adjustment for covariates (OR = 0.417, 95% CI: 0.188-0.926, P = 0.032).

**Conclusion:**

Rs1004467, rs4919687, rs10786712 of CYP17A1 gene are associated with CAD in Han population of China. The TT genotype of rs10786712 could be a protective genetic marker of CAD in men. The CC genotype of rs1004467 and the AA genotype of rs4919687 could be risk genetic markers of CAD in women. However, large sample size study including other SNPs of CYP17A1 should be performed in future studies.

## Introduction

Coronary artery disease (CAD) is a complex multifactorial disorder resulting from several susceptibility genes and multiple environmental determinants [1,2]. Recently, genetic basis of CAD has gained considerable interest [3], heritable factors accounted for 40%-60% in occurrence and development of CAD [4]. Various genes have been shown to be associated with CAD [5,6]. Some large-scale association studies have identified many common, uncommon and functional variants for CAD [7,8]. The CYP17A1 gene, located on chromosome 10q24.3, is mainly expressed in the adrenal glands and gonads. This gene encodes a member of enzymes of the cytochrome P450 superfamily. The cytochrome P450 proteins are monooxygenases and responsible for not only the metabolism of xenobiotics but also a host of endogenous substance whose metabolites have critical roles in the maintenance of cardiovascular health [9,10]. Mounting evidences have demonstrated that CYP enzymes are involved in the pathogenesis of CAD. For example, the CYP8A1 predominantly in vascular endothelial and smooth muscle cells, and acts mainly as an enzyme that converts prostaglandin H2 (PGH2) into prostacyclin (PGI2), some studies suggested that gene polymorphisms of CYP8A1 were associated with cardiovascular risk [11]. In addition, CYP1A1, CYP1A2 (metabolize tobacco polycyclic aromatic hydrocarbons and aromatic amines during smoking) [12,13], CYP2C8, CYP2J2 ([EET] synthesis) [14,15], CYP11B2 (aldosterone synthesis) [16], CYP17, and CYP19 (synthesis of sex hormones) [17], have been demonstrated to be associated with CAD.

In humans, CYP17A1 gene is responsible for the synthesis of P450c17 proteins, which is a key enzyme in the steroidogenic pathway. CYP17A1 genetic mutations affect the synthesis of steroids, which are the precursors of sex hormones. Some evidences have indicated that the levels of sex hormones can affect the development of cardiovascular and cerebrovascular diseases [18].

Recently, two large-scale association analyses identified 13 new susceptibility loci for CAD including CYP17A1 gene [19,20]. Adam S Butterworth et al. [21] selected 15596 patients with CAD and 34992 controls to examine 2100 genes including 49094 genetic variations, and suggested that CYP17A1 gene is one of the susceptibility genes for CAD. However, the relation between CYP17A1 gene and CAD in Chinese population remains unclear. Therefore, in the present study, we aimed to assess the association between the polymorphisms of CYP17A1 and CAD in Chinese Han population.

## Methods

### Ethical approval of the study protocol

This study was conducted according to the standards of the Declaration of Helsinki, and was approved by the Ethics Committee of the First Affiliated Hospital of Xinjiang Medical University (Xinjiang, China). Written informed consent was obtained from each participant, and explicitly provided permission for DNA analyses as well as collection of relevant clinical data.

### Subjects

Study population was from a Han population who lived in the Xinjiang Uygur Autonomous Region of China, all subjects attended as inpatients and had a differential diagnosis for chest pain encountered in the Cardiac Catheterization Laboratory of First Affiliated Hospital of Xinjiang Medical University from 2007 to 2013. Approximately 3000 patients undergo coronary angiography every year and we selected almost 1000 Han CAD patients and 1000 healthy persons diagnosed by angiography from 2007 to 2013. Highly skilled physicians were undertaken the coronary angiography using the Judkins approach. Least two experienced imaging specialists were interpreted the findings of coronary angiography, finally, the diagnosis of CAD was made according to the angiography report. All CAD groups defined as the presence of at least one significant coronary artery stenosis of > 50% luminal diameter. Patients with congenital heart disease, multiple organ failure syndrome, malignancy or chronic inflammatory disease were excluded. According to the exclusion criteria, 124 people were excluded from the 1000 CAD patients. Each control subject also underwent a coronary angiogram and did not show coronary artery stenosis. These individuals had no electrocardiographic signs of CAD, regional wall motion abnormalities, and no relevant valvular abnormalities in echocardiograms. Control subjects with CAD and any neoplasm, cardiomyopathy or severe illness limiting life expectance or refusing consent were excluded, according to the exclusion criteria, 82 people were excluded from the 1000 healthy persons. Finally, to ensure matching for age and gender, we selected 490 patients and 507 healthy persons from the 876 CAD patients and 918 healthy persons. Some of the controls have hypertension, and diabetes mellitus, which means control group expose to the same risk factors of CAD while the results of coronary angiogram are normal. The diagnosis of hypertension was established if patients were on antihypertensive medication or if the mean of 3 measurements of systolic blood pressure (SBP) at least 140 mmHg, and/or diastolic blood pressure (DBP) at least 90 mmHg, or a previous diagnosis of hypertension and the use of antihypertensive medication; Diabetes mellitus was defined on the basis of the American Diabetes Association [22]. In addition, individuals with fasting plasma glucose > 7.0 mmol/L or with a history of diabetes or treatment with hypoglycemic agent were considered diabetic. Smoking was classified as smokers (including current or ex-smokers) or non-smokers.

### Blood collection and DNA extraction

Before cardiac catheterization, 5 ml of fasting venous blood drawn by venipuncture in the Cardiac Catheterization Laboratory were taken from all participants. The blood samples were collected into tubes containing ethylene diamine tetraacetic acid (EDTA), and centrifuged at 4000 × g for 5 min to separate the plasma content (including plasma, serum and blood cells). Genomic DNA was extracted from the peripheral leukocytes using standard phenol-chloroform method [23]. The DNA samples were stored at −80°C for future analysis. Before genetic analysis, the final concentration of the DNA was diluted to 50 ng/μL.

### Genotyping

There are 662 SNPs for the human CYP17A1 gene listed in the National Center for Biotechnology Information SNP database (http://www.ncbi.nlm.nih.gov/SNP). Using Haploview 4.2 software and International HapMap Project website phase I &II database (http://www.hapmap.org), we obtained five tag SNPs (SNP1: rs4919686, SNP2: rs1004467, SNP3: rs4919687, SNP4: rs10786712, SNP5: rs2486758) by using minor allele frequency (MAF) ≥ 0.05 and linkage disequilibrium patterns with r^2^ ≥ 0.8 as a cut off. Genotyping in the present case–control study was confirmed by the TaqMan SNP Genotyping Assay (Applied Biosystems, Foster City, CA, USA). The primers and probes used in the TaqMan SNP Genotyping Assays were chosen based on information available at the ABI Web site (http://myscience.appliedbiosystems.com). Thermal cycling was done using the Applied Biosystems 7900HT Fast Real-Time PCR System. Plates were read on the sequence detection systems (SDS) automation controller software v2.3 (ABI). PCR amplification was performed using 2.5 μL of TaqMan Universal Master Mix, 0.15 μL probes and 1.85 ddH_2_O in a 6-μL final reaction volume containing 1 μL DNA. The thermal cycling conditions were as follows: 50°C for 2 min; 95°C for 10 min; 50 cycles of 95°C for 15 s; and 60°C for 1 min. Thermal cycling was performed using the Sequence Detection Systems (SDS) automation controller software v2.3 (ABI).

### Biochemical analysis

Serum concentrations of glucose (Glu), total cholesterol (TC), triglyceride (TG), high-density lipoprotein cholesterol (HDL-C), low-density lipoprotein cholesterol (LDL-C), blood urea nitrogen (BUN), creatinine (Cr) and uric acid (UA) were measured using standard methods in the Central Laboratory of First Affiliated Hospital of Xinjiang Medical University as described previously [24-26].

### Statistical analysis

Data analysis was carried out using the computer software Statistical Package for Social Sciences SPSS 17.0 for Windows (SPSS Institute, Chicago, USA). Hardy-Weinberg equilibrium was assessed by χ^2^ analysis. Differences in measurement variables (e.g. age, BMI, TC, TG, HDL-C, LDL-C) were analyzed using means ± standard deviation (SD). The difference between the CAD and control groups was analyzed using an independent-sample t-test. Differences in frequencies of smoking, drinking, hypertension, diabetes mellitus, and CYP17A1 genotypes were analyzed using χ^2^ test or Fisher’s exact test while appropriate. *P*-value < 0.05 was considered statistically significant. Logistic regression analyses with effect ratios (odds ratio [OR] and 95% CI) were used to assess contribution of major risk factors. Statistical significance was established at P < 0.05. Based on the genotype data of the genetic variations, we performed linkage disequilibrium (LD) analysis and haplotype-based case–control analysis, using the expectation maximization (EM) algorithm [27] and the software SHEsis (http://analysis.bio-x.cn/SHEsisMain.htm). The pairwise linkage disequilibrium analysis was performed using five SNP pairs. We used∣D'∣values of > 0.5 to assign SNP locations to one haplotype block. Single nucleotide polymorphisms with an r^2^ value of < 0.5 were selected as tagged. In the haplotype-based case–control analysis, haplotypes with a frequency of < 0.03 were excluded. The frequency distribution of the haplotypes was calculated by performing a permutation test using the bootstrap method.

## Results

Table 1 shows demographic and clinical characteristics of the study participants. There was no significant difference in age between CAD patients and control subjects. It means the study was an age-matched case–control study. For total subjects, men, and women participants, there was no significant difference in the following variables between the CAD patients and the control participants: hypertension, smoking, drinking, diastolic blood pressure (DBP), and serum concentration of total cholesterol, low-density lipoprotein cholesterol, and uric acid. The incidence of diabetes, and the plasma concentration of glucose, creatinine was significantly higher in subjects with CAD than in the controls. For total participants, the following values were significantly higher for the CAD patients as compared to the control subjects: systolic blood pressure (SBP), the plasma concentration of triglyceride, high-density lipoprotein cholesterol. And the plasma concentration of HDL was significantly lower for patients with CAD than for control participants. For women, the systolic blood pressure (SBP), triglyceride, were significantly higher for patients with CAD than for control participants.

**Table 1:** **Demographic and clinical characteristics of study participants**

	**Total**			**Men**			**Women**		
	**CAD**	**Control**	**P value**	**CAD**	**Control**	**P value**	**CAD**	**Control**	**P value**
Number(n)	490	507		278	263		212	244	
Age, mean (SD)	61.94 (9.97)	61.20 (10.07)	0.242	60.65 (11.11)	59.70 (11.32)	0.326	63.63 (7.95)	62.81 (8.23)	0.280
EH, n (%)	236 (48.2)	219 (43.2)	0.115	119 (42.8)	106 (40.3)	0.555	117 (55.2)	113 (46.3)	0.059
Diabetes, n (%)	102 (20.8)	40 (7.9)	<0.001	55 (19.8)	23 (8.7)	<0.001	47 (22.2)	17 (7.0)	<0.001
Smoking, n (%)	63 (12.9)	50 (9.9)	0.136	60 (21.6)	49 (18.6)	0.392	3 (1.4)	1 (4)	0.519
Drinking, n (%)	55 (11.2)	43 (8.5)	0.146	54 (19.4)	43 (16.3)	0.371	1 (5)	0	0.994
BMI, mean (SD)	25.85 (3.41)	25.48 (3.26)	0.076	26.41 (3.35)	25.72 (3.37)	0.018	25.26 (3.39)	25.16 (3.10)	0.753
SBP, mean (SD)	138.74 (26.35)	135.49 (24.09)	0.045	136.44 (25.77)	134.82 (23.11)	0.448	141.84 (26.87)	136.20 (25.13)	0.024
DBP, mean (SD)	85.78 (18.10)	84.18 (15.66)	0.143	85.48 (18.42)	84.52 (15.94)	0.521	86.17 (17.71)	83.81 (15.38)	0.136
Glu, mean (SD)	6.28 (2.69)	5.55 (1.73)	<0.001	6.21 (2.77)	5.62 (1.92)	0.004	6.37 (2.57)	5.48 (1.50)	< 0.001
TG, mean (SD)	2.24 (2.13)	1.90 (1.48)	0.005	2.30 (2.10)	2.04 (1.57)	0.110	2.15 (2.17)	1.75 (1.37)	0.026
TC, mean (SD)	4.25 (1.16)	4.33 (1.02)	0.306	4.06 (0.99)	4.18 (1.04)	0.160	4.51 (1.30)	4.48 (0.97)	0.777
HDL, mean (SD)	1.08 (0.33)	1.13 (0.32)	0.013	1.01 (0.28)	1.05 (0.29)	0.148	1.16 (0.38)	1.22 (0.33)	0.104
LDL, mean (SD)	2.51 (0.94)	2.55 (0.86)	0.457	2.43 (0.91)	2.52 (0.89)	0.220	2.80 (2.46)	2.57 (0.82)	0.212
UA, mean (SD)	320.94 (94.17)	321.07 (82.94)	0.982	344.51 (91.79)	348.25 (75.07)	0.609	289.55 (88.13)	291.72 (81.14)	0.788
Cr, mean (SD)	77.79 (33.00)	72.21 (17.82)	0.001	84.09 (33.96)	79.15 (16.84)	0.033	69.39 (29.73)	64.71 (15.71)	0.045
BUN, mean (SD)	5.38 (1.96)	5.29 (1.86)	0.446	5.54 (1.68)	5.51 (1.68)	0.822	5.17 (2.27)	5.05 (2.01)	0.562

Data are presented as mean ± SD or n (%). Continuous variables are expressed as mean ± SD. Categorical variables are expressed as percentages. MI, body mass index; BUN, blood urea nitrogen; Cr, creatinine; DBP, diastolic blood pressure; DM, diabetes mellitus; Glu, glucose; TG, triglyceride; TC, total cholesterol; HDL, high density lipoprotein; LDL, low density lipoprotein; EH, essential hypertension; SBP, systolic blood pressure; UA, uric acid.

The P value of the continuous variables was calculated by the Independent t-test. The P value of the categorical variables was calculated by Fisher’s exact test.

Table 2 shows the distribution of genotypes and alleles of SNP1, SNP2, SNP3, SNP4 and SNP5 for the CYP17A1 gene. The genotype distributions for each SNP were in good agreement with the predicted Hardy–Weinberg equilibrium values (data not shown). For total, men, and women subjects, the distribution of SNP1 (rs4919686) and SNP5 (rs2486758) genotypes, dominant model, recessive model, and additive model did not show a significant difference between CAD and control participants (*P* > 0.05, respectively). For total participants, the other three SNPs (SNP2: rs1004467, SNP3: rs4919687, SNP4: rs10786712) genotypes, dominant model, recessive model, additive model, and allele frequency also did not show a significant difference between the CAD patients and the control subjects (P > 0.05 respectively). For men subjects, distribution of rs10786712 recessive model (CC + CT *vs.* TT) showed difference between CAD and control subjects (P = 0.016), and the recessive model was significantly lower in subjects with CAD than in controls (75.7% *vs.* 66.1%). For women participants, the dominant model (CC + CT *vs.* TT) of rs1004467 showed difference between CAD and control subjects (P = 0.038), and the dominant model was significantly higher in CAD patients than in control participants (76.6% *vs.* 67.8%). The distribution of the recessive model (AG + GG *vs.* AA) of rs4919687 was significantly higher in patients with CAD than in control participants (89.8% *vs.* 95.5%), and showed a significant difference between CAD and control subjects (P = 0.019).

**Table 2:** **Genotype and Allele distributions in patients with CAD and control participants**

**Variants**		**Total**			**Man**			**Woman**		
		**CAD, n (%)**	**Control, n (%)**	**P value**	**CAD n (%)**	**Control n (%)**	**P value**	**CAD n (%)**	**Control n (%)**	**P value**
Rs4919686 (SNP1)										
Genotyping	AA	365 (76.2)	356 (77.1)	0.710	205 (75.6)	181 (76.1)	0.836	160 (76.9)	175 (78.1)	0.811
	AC	110 (23.0)	100 (21.6)		63 (23.2)	53 (22.3)		47 (22.6)	47 (21.0)	
	CC	4 (0.8)	6 (1.3)		3 (1.1)	4 (1.7)		1 (0.5)	2 (0.9)	
Recessive model	CC	4 (0.8)	6 (1.3)	0.707	3 (1.1)	4 (1.7)	0.863	1 (0.5)	2 (0.9)	1
	AA + AC	475 (99.2)	456 (98.7)		268 (98.9)	234 (98.3)		207 (99.5)	222 (99.1)	
Dominant model	AA	365 (76.2)	356 (77.1)	0.756	205 (75.6)	181 (76.1)	0.915	160 (76.9)	175 (78.1)	0.765
	AC + CC	114 (23.8)	106 (22.9)		66 (24.4)	57 (23.9)		48 (23.1)	49 (21.9)	
Additive model	AC	110 (23.0)	100 (21.6)	0.627	63 (23.2)	53 (22.3)	0.793	47 (22.6)	47 (21.0)	0.685
	AA + CC	369 (77.0)	362 (78.4)		208 (76.8)	185 (77.7)		161 (77.4)	177 (79.0)	
Allele	A	840 (87.7)	812 (87.9)	0.897	473 (87.3)	415 (87.2)	0.968	367 (88.2)	397 (88.6)	0.856
	C	118 (12.3)	112 (12.1)		69 (12.7)	61 (12.8)		49 (11.8)	51 (11.4)	
Rs1004467 (SNP2)										
Genotyping	CC	86 (17.8)	89 (17.5)	0.993	41 (14.7)	45 (17.1)	0.240	45 (22.0)	44 (18.0)	0.106
	CT	250 (51.8)	264 (52.0)		138 (49.6)	142 (54.0)		112 (54.6)	122 (49.8)	
	TT	147 (30.4)	155 (30.5)		99 (35.6)	76 (28.9)		48 (23.4)	79 (32.2)	
Recessive model	CC	86 (17.8)	89 (17.5)	0.906	41 (14.7)	45 (17.1)	0.453	45 (22.0)	44 (18.0)	0.290
	CT + TT	397 (82.2)	419 (82.5)		237 (85.3)	218 (82.9)		160 (78.0)	201 (82.0)	
Dominant model	TT	147 (30.4)	155 (30.5)	0.979	99 (35.6)	76 (28.9)	0.095	48 (23.4)	79 (32.2)	0.038
	CC + CT	336 (69.6)	353 (69.5)		179 (64.4)	187 (71.1)		157 (76.6)	166 (67.8)	
Additive model	CT	250 (51.8)	264 (52.0)	0.948	138 (49.6)	142 (54.0)	0.311	112 (54.6)	122 (49.8)	0.306
	CC + TT	233 (48.2)	244 (48.0)		140 (50.4)	121 (46.0)		93 (45.4)	123 (50.2)	
Allele	C	422 (43.7)	442 (43.5)	0.935	220 (39.6)	232 (44.1)	0.130	202 (49.3)	210 (42.9)	0.055
	T	544 (56.3)	574 (56.5)		336 (60.4)	294 (55.9)		208 (50.7)	280 (57.1)	
Rs4919687 (SNP3)										
Genotyping	AA	30 (6.3)	24 (4.7)	0.551	9 (3.3)	13 (5.0)	0.538	21 (10.2)	11 (4.5)	0.063
	AG	155 (32.4)	171 (33.8)		93 (33.9)	93 (35.5)		62 (30.2)	78 (32.0)	
	GG	294 (61.4)	311 (61.5)		172 (62.8)	156 (59.5)		122 (59.5)	155 (63.5)	
Recessive model	AA	30 (6.3)	24 (4.7)	0.295	9 (3.3)	13 (5.0)	0.328	21 (10.2)	11 (4.5)	0.019
	AG + GG	449 (93.7)	482 (95.3)		265 (96.7)	249 (95.0)		184 (89.8)	233 (95.5)	
Dominant model	GG	294 (61.4)	311 (61.5)	0.978	172 (62.8)	156 (59.5)	0.443	122 (59.5)	155 (63.5)	0.384
	AA + AG	185(38.6)	195 (38.5)		102 (37.2)	106 (40.5)		83 (40.5)	89 (36.5)	
Additive model	AG	155 (32.4)	171 (33.8)	0.632	93 (33.9)	93 (35.5)	0.705	62 (30.2)	78 (32.0)	0.695
	AA + GG	324 (67.6)	335 (66.2)		181 (66.1)	169 (64.5)		143 (69.8)	166 (68.0)	
Allele	A	215 (22.4)	219 (21.6)	0.668	111 (20.3)	119 (22.7)	0.328	104 (25.4)	100 (20.5)	0.083
	G	743 (77.6)	793 (78.4)		437 (79.7)	405 (77.3)		306 (74.6)	388 (79.5)	
Rs10786712 (SNP4)										
Genotyping	CC	114 (23.7)	96 (20.8)	0.407	64 (23.5)	51 (21.3)	0.055	50 (23.9)	45 (20.2)	0.264
	CT	239 (49.7)	228 (49.4)		142 (52.2)	107 (44.8)		97 (46.4)	121 (54.3)	
	TT	128 (26.6)	138 (29.9)		66 (24.3)	81 (33.9)		62 (29.7)	57 (25.6)	
Recessive model	TT	128 (26.6)	138 (29.9)	0.266	66 (24.3)	81 (33.9)	0.016	62 (29.7)	57 (25.6)	0.340
	CC + CT	353 (73.4)	324 (70.1)		206 (75.7)	158 (66.1)		147 (70.3)	166 (74.4)	
Dominant model	CC	114 (23.7)	96 (20.8)	0.281	64 (23.5)	51 (21.3)	0.554	50 (23.9)	45 (20.2)	0.348
	CT + TT	367 (76.3)	366 (79.2)		208 (76.5)	188 (78.7)		159 (76.1)	178 (79.8)	
Additive model	CT	239 (49.7)	228 (49.4)	0.917	142 (52.2)	107 (44.8)	0.093	97 (46.4)	121 (54.3)	0.103
	CC + TT	242 (50.3)	234 (50.6)		130 (47.8)	132 (55.2)		112 (53.6)	102 (45.7)	
Allele	C	467(48.5)	420 (45.5)	0.179	270 (49.6)	209 (43.7)	0.059	197 (47.1)	211 (47.3)	0.958
	T	495 (51.5)	504 (54.5)		274 (50.4)	269 (56.3)		221 (52.9)	235 (52.7)	
Rs2486758 (SNP5)										
Genotyping	CC	14 (2.9)	16 (3.2)	0.950	9 (3.3)	10 (3.8)	0.912	5 (2.4)	6 (2.5)	0.983
	CT	162 (33.3)	164 (32.8)		95 (34.5)	87 (33.3)		67 (31.6)	77 (32.2)	
	TT	311 (63.9)	320 (64.0)		171 (62.2)	164 (62.8)		140 (66.0)	156 (65.3)	
Recessive model	CC	14 (2.9)	16 (3.2)	0.766	9 (3.3)	10 (3.8)	0.727	5 (2.4)	6 (2.5)	0.917
	CT + TT	473 (97.1)	484 (96.8)		266 (96.7)	251 (96.2)		207 (97.6)	233 (97.5)	
Dominant model	TT	311 (63.9)	320 (64.0)	0.964	171 (62.2)	164 (62.8)	0.876	140 (66.0)	156 (65.3)	0.864
	CC + CT	176 (36.1)	180 (36.0)		104 (37.8)	97 (37.2)		72 (34.0)	83 (34.7)	
Additive model	CT	162 (33.3)	164 (32.8)	0.877	95 (34.5)	87 (33.3)	0.767	67 (31.6)	77 (32.2)	0.889
	CC + TT	325 (66.7)	336 (67.2)		180 (65.5)	174 (66.7)		145 (68.4)	162 (67.8)	
Allele	C	190 (19.5)	196 (19.6)	0.959	113 (20.5)	107 (20.1%	0.985	77 (18.2)	89 (18.6)	0.859
	T	784 (80.5)	804 (80.4)		437 (79.5)	415 (79.5)		347 (81.8)	389 (81.4)	

CAD, Coronary artery disease; N, number of participants; SNP, single-nucleotide polymorphism.

Tables 3, 4, and 5 showed the multivariable logistic regression analyses done with the following variables: prevalence of conventional risk factors for CAD including hypertension, diabetes, smoking and drinking, and plasma concentration of blood glucose, TG, Cr, and SBP. For women (Table 3), after multivariate adjustment, rs1004467 remains significantly association with CAD in dominant model (OR = 1.623, 95%CI: 1.023-2.576, P = 0.040); rs4919687 (Table 4) remains significantly association with CAD (OR = 0.417, 95%CI: 0.188-0.926, P = 0.032) in recessive model. For men (Table 5), the significant difference of rs10786712 (OR = 1.644, 95% confidence interval [CI]:1.087-2.488, P = 0.019) was retained after adjustment of the major confounding factors for CAD in recessive model.

**Table 3:** **Multiple logistic regression analysis for CAD patients and control subjects (rs1004467)**

	**Total**			**Men**			**Woman**		
	**OR**	**95% CI**	**P**	**OR**	**95% CI**	**P**	**OR**	**95% CI**	**P**
Dominant model (CC + CT vs TT)	1.024	0.766- 1.370	0.872	0.731	0.498- 1.075	0.112	1.623	1.023- 2.576	0.040
Hypertension	0.988	0.729- 1.340	0.939	0.955	0.635- 1.436	0.825	1.082	0.670- 1.748	0.747
Diabetes	2.485	1.619- 3.812	< 0.001	2.246	1.248- 4.044	0.007	2.957	1.556- 5.619	0.001
Smoking	1.490	0.827- 2.676	0.184	1.381	0.748- 2.549	0.303	2.291	0.180- 29.077	0.523
Drinking,	0.908	0.485- 1.703	0.764	0.941	0.493- 1.796	0.845	0	0	1
Glucose	1.147	1.058- 1.243	0.001	1.107	0.991- 1.235	0.071	1.198	1.065- 1.347	0.003
triglyceride	1.088	1.055- 1.177	0.038	1.060	0.960- 1.171	0.249	1.138	0.988- 1.310	0.072
Creatinine	1.099	1.002- 1.005	0.011	1.009	1.000- 1.018	0.046	1.004	0.991- 1.016	0.567
SBP	1.003	0.997- 1.009	0.271	1.001	0.993- 1.009	0.851	1.006	0.997- 1.015	0.191

CAD, Coronary artery disease; OR, odds ratios; 95%CI, 95% confidence intervals; SBP, systolic blood pressure.

**Table 4:** **Multiple logistic regression analysis for CAD patients and control subjects (rs4919687)**

	**Total**			**Men**			**Woman**		
	**OR**	**95% CI**	**P**	**OR**	**95% CI**	**P**	**OR**	**95% CI**	**P**
Recessive model (AG + GG vs AA)	0.673	0.372- 1.217	0.190	1.324	0.512- 3.428	0.563	0.417	0.188- 0.926	0.032
Hypertension	1.001	0.738- 1.358	0.996	0.958	0.637- 1.442	0.838	1.155	0.717- 1.862	0.55
Diabetes	2.484	1.618- 3.814	< 0.001	2.370	1.310- 4.289	0.004	2.857	1.508- 5.411	0.001
Smoking	1.546	0.853- 2.803	0.151	1.455	0.783- 2.701	0.235	2.959	2.33- 37.579	0.403
Drinking,	0.861	0.455- 1.629	0.645	0.864	0.449- 1.662	0.662	0	0	1
Glucose	1.154	1.063- 1.253	0.001	1.094	0.979- 1.223	0.114	1.218	1.080- 1.373	0.001
triglyceride	1.096	1.012- 1.187	0.024	1.064	0.963- 1.175	0.224	1.154	1.003- 1.327	0.045
Creatinine	1.009	1.002- 1.015	0.013	1.009	1.000- 1.018	0.054	1.003	0.991- 1.016	0.606
SBP	1.003	0.997- 1.009	0.272	1.001	0.993- 1.009	0.853	1.005	0.996- 1.014	0.246

CAD, Coronary artery disease; OR, odds ratios; 95%CI, 95% confidence intervals; SBP, systolic blood pressure.

**Table 5:** **Multiple logistic regression analysis for CAD patients and control subjects (rs10786712)**

	**Total**			**Men**			**Woman**		
	**OR**	**95% CI**	**P**	**OR**	**95% CI**	**P**	**OR**	**95% CI**	**P**
Recessive model (CC + CT vs TT)	1.193	0.879- 1.619	0.259	1.644	1.087- 2.488	0.019	0.786	0.495- 1.248	0.308
Hypertension	1.010	0.742- 1.377	0.947	0.996	0.655- 1.513	0.985	1.117	0.691- 1.805	0.651
Diabetes	2.305	1.481- 3.587	< 0.001	2.458	1.313- 4.600	0.005	2.433	1.278- 4.630	0.007
Smoking	1.523	0.833-2.785	0.172	1.421	0.756- 2.669	0.275	2.637	0.210- 33.171	0.453
Drinking,	0.825	0.434- 1.567	0.556	0.835	0.431- 1.618	0.593	0	0	1
Glucose	1.171	1.075- 1.276	< 0.001	1.111	0.988- 1.250	0.079	1.227	1.084- 1.388	0.001
triglyceride	1.082	0.999- 1.571	0.052	1.062	0.960- 1.175	0.243	1.134	0.986- 1.304	0.077
Creatinine	1.009	1.001- 1.016	0.010	1.010	1.000-1.019	0.046	1.005	0.993- 1.017	0.433
SBP	1.002	0.996- 1.008	0.492	0.999	0.990- 1.007	0.795	1.005	0.996- 1.014	0.281

CAD, Coronary artery disease; OR, odds ratios; 95%CI, 95% confidence intervals; SBP, systolic blood pressure.

Table 6 shows patterns of linkage disequilibrium (LD) analysis in the CYP17A1. All 5 SNPs are located in one haplotype block for∣D'∣values were beyond 0.5, and all of the r^2^ values were below 0.5. Because the∣D'∣for SNP2-SNP3 was < 0.5, this meant that SNP2 and SNP3 could not be used to simultaneously construct haplotypes. As the minor allele frequency of SNP3 was larger than that of SNP2, we constructed haplotypes using SNP1, SNP3, SNP4, and SNP5.

**Table 6:** **Pairwise linkage disequilibrium for five SNPs**

**∣D'∣ values**						
r^2^ values		SNP1	SNP2	SNP3	SNP4	SNP5
	SNP1		0.575	0.911	1.000	0.913
	SNP2	0.035		0.470	0.567	0.630
	SNP3	0.407	0.049		0.874	0.723
	SNP4	0.123	0.219	0.194		0.926
	SNP5	0.028	0.074	0.036	0.236	

∣D'∣ above the diagonal and r^2^ below the diagonal.

Table 7 shows the result of haplotype analysis. In the haplotype-based case–control analysis, haplotypes were established through different combinations of the 4 SNPs. For total, including men and women, the overall distribution of haplotypes were no significantly different between the CAD patients and the control subjects. For men, the frequencies of the C–T, G–C–T, A–C–T, and A–G–C–T haplotypes respectively established by SNP4–SNP5, SNP3–SNP4–SNP5, SNP1–SNP4–SNP5, and SNP1–SNP3–SNP4–SNP5 were significantly higher for the CAD patients as compared to the control subjects (P = 0.047, P = 0.048, P = 0.040, and P = 0.039, respectively). The frequencies of the A–A–T, A–T–T, and A–A–T–T haplotypes respectively established by SNP1–SNP3–SNP5, SNP1–SNP4–SNP5, and SNP1–SNP3–SNP4–SNP5 were lower for CAD patients than for control participants (P = 0.040, P = 0.031, P = 0.033, respectively). For women, the frequency of the A–T, A–A–T, A–A–T, and A–A–T–T haplotypes established by SNP3–SNP4, SNP1–SNP3–SNP4, SNP1–SNP3–SNP5, and SNP1–SNP3–SNP4–SNP5 respectively were significantly higher for the CAD patients as compared to the control subjects (P = 0.041, P = 0.034, P = 0.035, and P = 0.045, respectively).

**Table 7:** **Haplotype analysis in patients with CAD and in control subjects**

**Haplotype**	**No. 1**	**No. 2**	**No. 3**	**No. 4**	**Overall P value**			**Frequency in total**			**Frequency in man**			**Frequency in woman**		
					**Total**	**Man**	**Woman**	**CAD**	**Control**	**P value**	**CAD**	**Control**	**P value**	**CAD**	**Control**	**P value**
		SNP3		SNP5	0.877	0.340	0.105									
H1		A		T				0.217	0.206	0.617	0.188	0.222	0.190	0.249	0.189	0.041
H2		G		C				0.183	0.182	0.985	0.188	0.196	0.754	0.170	0.167	0.951
H3		G		T				0.591	0.597	0.686	0.609	0.570	0.182	0.573	0.627	0.074
			SNP4	SNP5	0.399	0.099	0.964									
H1			C	C				0.192	0.186	0.793	0.201	0.195	0.857	0.180	0.177	0.985
H2			C	T				0.294	0.268	0.235	0.299	0.241	0.047	0.288	0.296	0.787
H3			T	T				0.509	0.535	0.201	0.494	0.550	0.055	0.528	0.520	0.854
	SNP1	SNP3	SNP4		0.612	0.167	0.155									
H1	A	A	T					0.094	0.094	0.981	0.070	0.104	0.053	0.127	0.082	0.034
H2	A	G	C					0.471	0.446	0.280	0.487	0.436	0.102	0.449	0.456	0.834
H3	A	G	T					0.299	0.327	0.192	0.307	0.324	0.552	0.290	0.331	0.189
	SNP1	SNP3		SNP5	0.961	0.225	0.154									
H1	A	A		T				0.098	0.096	0.933	0.067	0.103	0.040	0.135	0.088	0.035
H2	A	G		C				0.186	0.181	0.795	0.192	0.194	0.972	0.173	0.167	0.853
H3	C	A		T				0.116	0.109	0.701	0.119	0.117	0.893	0.110	0.102	0.742
	SNP1		SNP4	SNP5	0.415	0.117	0.986									
H1	A		C	T				0.295	0.268	0.225	0.302	0.242	0.040	0.286	0.296	0.733
H2	A		T	T				0.385	0.419	0.104	0.364	0.427	0.031	0.415	0.411	0.953
H3	C		T	T				0.122	0.115	0.668	0.127	0.121	0.830	0.114	0.109	0.830
		SNP3	SNP4	SNP5	0.463	0.211	0.282									
H1		A	T	T				0.211	0.205	0.776	0.190	0.221	0.219	0.239	0.187	0.071
H2		G	C	C				0.180	0.177	0.905	0.190	0.189	0.968	0.165	0.164	0.976
H3		G	C	T				0.291	0.266	0.239	0.297	0.241	0.048	0.284	0.292	0.761
	SNP1	SNP3	SNP4	SNP5	0.577	0.098	0.302									
H1	A	A	T	T				0.092	0.094	0.878	0.066	0.103	0.033	0.127	0.084	0.045
H2	A	G	C	C				0.181	0.177	0.851	0.191	0.189	0.949	0.169	0.165	0.913
H3	A	G	C	T				0.292	0.226	0.221	0.300	0.243	0.039	0.280	0.290	0.693
H4	A	G	T	T				0.294	0.326	0.124	0.297	0.324	0.360	0.291	0.329	0.213
H5	C	A	T	T				0.117	0.110	0.673	0.121	0.116	0.817	0.110	0.103	0.753

CAD, Coronary artery disease; haplotype with frequencies >0.03 were estimated using SHEsis software; P value was calculated by permutation test using the bootstrap method; SNP, single-nucleotide polymorphism.

## Discussion

Relationship between CYP genetic polymorphism and cardiovascular disease (CVD) has been established [10]. In our study, we found the variations in CYP17A1 gene was associated with CAD in a Han population of China, even after multivariate adjustment, the association still maintained This is the first study to reveal the relation between CAD and CYP17A1 gene in Chinese population.

The pathogenesis of CAD includes the disorders of lipoprotein metabolism [28,29], disturbance of blood coagulation and fibrinolytic system [30,31], insulin resistance or diabetes [32,33], hypertension [34,35], and the impairment and inflammation of vascular endothelium [36-38]. As previously mentioned, P450c17proteins is an important enzyme that catalyzes the formation of all endogenous androgens. Therefore, CYP17A1 genetic mutations can loss of the enzyme activity of P450c17 and potentially reduce androgen biosynthesis.

In recent years, many clinical studies showed that testosterone levels play an important role in the progress of CAD among elderly men [39], whereas lower testosterone levels promote CAD [40]. In addition, some evidences have indicated that testosterone also was related to the risk factors of CAD. For example, numerous studies have confirmed that the dysfunction of vascular endothelial as a key mechanism of occurrence and development of CAD [36-38], the levels of physiological testosterone can promote the endothelial cells release of nitric oxide (NO) through improve the vascular endothelial function, and low levels of testosterone can decrease the vascular endothelial function and promote the occurrence of CAD [41]. The disorders of lipoprotein metabolism also one of primary mechanisms of CAD [28,29], research shows there was a positive correlation between the levels of testosterone and HDL-C, and a negatively correlated between the levels of testosterone and the TG, TC, LDL-C and VLDL. Malkin et al. also confirmed that the low levels of testosterone can lead to lipid disorders, and supplement testosterone can correct dyslipidemia [42]. Insulin resistance and diabetes are the significant independent risk factors for CAD [32,33]. Selvin et al. indicated that there is a relationship between diabetes and the low free or low bioactive testosterone levels [43]. Blood coagulation and fibrinolytic system is the important mechanism of the CAD [30,31], physiological levels of testosterone can improve the function of endothelial cells [44], low levels of testosterone can increase the proteins related to clotting factor VIII, causing endothelial dysfunction and vascular inflammatory reaction, finally, platelet adhesion in the damaged blood vessels, resulting in the incidence of CAD. Androgens serve as precursors to estrogens, so normal estrogen signaling is also dependent on CYP17A1. Estrogen plays a very important role in many other physiological and pathological process, such as mediation vasoconstriction, vascular endothelium repair and lipid metabolism, involves in glucose metabolism and insulin related signal transduction pathways, etc., which are directly or indirectly affect the function of cardiovascular system. Wellons et al. reported that early menopause is associated with an increased risk of CAD [45], as well as high levels of endogenous estrogen explain the low prevalence of CAD in premenopausal women [46]. It is worth noting that the protective value of sex hormones appears to be sex-specific, high levels of estrogen and oestrone in men are associated with an increased risk of and CAD [39].

In our study, we found that polymorphisms of CYP17A1 were associated with risk of CAD in a Han population. For rs10786712, in men, the recessive model (CC + CT vs TT) was significantly higher in control subjects than in CAD patients, after multivariate adjustment of confounding factors such as plasma concentration of TG, Glu, Cr, incidence of hypertension, diabetes, drinking, and smoking for CAD, the significant difference was retained. This indicated that the TT genotype might be protecting against for CAD in men. For rs1004467 and rs4919687, in women, the dominant model (rs1004467) and recessive model (rs4919687) were significantly higher in CAD subjects than in control participants, after multivariate adjustment of confounding factors, the significant difference was retained. This result indicated that CC genotype of rs1004467 and AA genotype of rs4919687 are risk factor for CAD in women.

In addition, we hypothesized that haplotype analysis would be useful for the assessment of association between haplotypes and CAD. For men, we found a susceptible haplotype [A-C-T (SNP1-SNP4-SNP5)], and a protective haplotype [A-T-T (SNP1-SNP4-SNP5)]. And these haplotypic analysis results were consistent with the genotypic analysis results of SNP4 (rs10786712) that the CC genotype confers risk and the TT genotype is protective. For women, significant differences were found for the frequency of occurrence of the haplotype (A-T of SNP3-SNP5, A-A-T of SNP1-SNP3-SNP4, A-A-T of SNP1-SNP3-SNP5, and A-A-T-T of SNP1-SNP3-SNP4-SNP5, respectively).

There are still several limitations must be mentioned in the present study. Firstly, we did not perform enzymatic LDL, which would decrease the mean and SD of the LDL results. Secondly, we did not collect the data on lipid-lowering drug levels and drug compliance. Finally, this study was limited by the relatively small sample size, it needs a large number of clinical samples and investigation of other SNPs of CYP17A1 in future studies.

## Conclusion

In conclusion, this is the first study to investigate the differences between the human CYP17A1 and CAD in Han population of China, and is the first haplotype-based case–control study and to correlations its association with CAD. Rs1004467, rs4919687, rs10786712 of CYP17A1gene are associated with CAD in Han population of China. The TT genotype of rs10786712 could be a protective genetic marker of CAD in men. The CC genotype of rs1004467 and the AA genotype of rs4919687 could be two risk genetic marker of CAD in women. However, large sample size study including other SNPs of CYP17A1 should be performed in future studies.
